# Female Sexual Dysfunction and Pelvic Floor Muscle Function Associated with Systemic Sclerosis: A Cross-Sectional Study

**DOI:** 10.3390/ijerph19010612

**Published:** 2022-01-05

**Authors:** Barbora Heřmánková, Maja Špiritović, Hana Šmucrová, Sabína Oreská, Hana Štorkánová, Martin Komarc, Karel Pavelka, Ladislav Šenolt, Jiří Vencovský, Radim Bečvář, Michal Tomčík

**Affiliations:** 1 Institute of Rheumatology, 128 00 Prague, Czech Republic; hermankova@revma.cz (B.H.); spiritovic@revma.cz (M.Š.); smucrova@revma.cz (H.Š.); oreska@revma.cz (S.O.); storkanova@revma.cz (H.Š.); pavelka@revma.cz (K.P.); senolt@revma.cz (L.Š.); vencovsky@revma.cz (J.V.); becvar@revma.cz (R.B.); 2 Department of Physiotherapy, Faculty of Physical Education and Sport, Charles University, 162 52 Prague, Czech Republic; 3 Department of Rheumatology, First Faculty of Medicine, Charles University, 121 08 Prague, Czech Republic; 4 Department of Methodology, Faculty of Physical Education and Sport, Charles University, 162 52 Prague, Czech Republic; komarc@volny.cz

**Keywords:** systemic sclerosis, sexual health, female sexual dysfunction, pelvic floor function, quality of life

## Abstract

Only a few studies have addressed sexual health in patients with systemic sclerosis (SSc). This study aimed to compare female sexual function and pelvic floor muscle function in SSc patients with healthy controls (HC) matched by age, and to identify the potential implications of clinical features on sexual function. Our cohort included 90 women with SSc and 90 HC aged 18–70 years that completed six well-established and validated questionnaires assessing sexual function (Brief Index of Sexual Function for Women, Female Sexual Function Index, Sexual Quality of Life Questionnaire–Female, Sexual Function Questionnaire) and pelvic floor function (Pelvic Floor Impact Questionnaire–Short Form 7 and Pelvic Organ Prolapse/Urinary Incontinence Sexual Questionnaire Short Form). Results from women with SSc and HC were contrasted and correlated with relevant clinical features. The prevalence of female sexual dysfunction was 73% in SSc patients (vs. 31% in HC). Women with SSc reported significantly worse pelvic floor function and sexual function than HC. Impaired sexual function was correlated with higher disease activity, the presence of dyspnea and interstitial lung disease, increased systemic inflammation, reduced physical activity, functional disability, more severe depression, more pronounced fatigue, and impaired quality of life. We demonstrate that sexual dysfunction is highly prevalent among women with SSc. This aspect of the disease deserves more attention both in clinical care and at the level of international research collaboration.

## 1. Introduction

Systemic sclerosis (scleroderma, SSc) is an immune-mediated connective tissue disease characterized by vasculopathy and tissue fibrosis of the skin and various internal organs. The development of multi-organ manifestations can lead to multiple clinical complications and significantly reduce quality of life [1] including one’s sexual life [2]. Physical and psychological consequences of SSc that may affect sexual function include skin tightening, Raynaud’s phenomenon, microstomia and other disfigurement, painful digital ulcers, muscle weakness, gastrointestinal symptoms, dyspnea, fatigue, and depression [3]. Moreover, women with SSc often experience discomfort or pain during intercourse attributable to vaginal tightness and dryness. Lubrication can be decreased due to vaginal mucosal changes [4] and secondary Sjögren’s syndrome, which is present in approximately 25% of SSc patients [5]. Furthermore, many symptomatic medications can inhibit sexual desire and function including diuretics, vasoactive substances, and antidepressants such as selective serotonin reuptake inhibitors [6]. However, despite these findings, only a limited number of studies have addressed sexual function in women with SSc.

The first study was conducted by Bhadauria et al. [7] in 1995, comparing sexual function between 60 female SSc patients and 23 female patients with rheumatoid arthritis or systemic lupus erythematosus matched by age and disease duration. They demonstrated that women with SSc experienced fewer and weaker orgasms than the controls. Another study [4] assessed 83 women with SSc, 57% of whom reported sexual problems. Neither of these two studies used validated questionnaires or included healthy controls (HC). 

Subsequently, Knafo et al. [8] compared sexual impairment in 39 women with diffuse cutaneous SSc (dcSSc) and 99 women with limited cutaneous SSc (lcSSc) to women with other chronic conditions including breast cancer, HIV-positivity, and gynecological cancer. Women with dcSSc reported significantly greater sexual impairment than breast cancer and HIV-positive patients. Moreover, worse sexual function was observed in patients with dcSSc compared to lcSSc [8]. The study by Impens et al. [6] evaluated the sexual activity of 101 female SSc patients and concluded that 59% of the patients remained sexually active. The lack of sexual activity was due to the absence of a partner, the partner’s health status, and personal choice. Only 17% of the patients listed SSc as a primary reason for sexual inactivity. Schouffoer et al. [9] compared sexual function and distress between 37 patients and 37 healthy women of similar age (±5 years). The FSFI total score and its subscores for lubrication, orgasm, arousal, and pain in SSc were significantly lower than in HC. Longer disease duration, greater depressive symptoms, and the use of antidepressants were associated with sexual dysfunction and distress [9]. These studies are limited by the absence of age-matched HC and a small sample size. 

Another study by Knafo et al. aimed to assess the association of body image dissatisfaction, pain, and sociodemographic variables with reduced sexual function in 117 women with SSc. Multivariate linear regression revealed the independent association of reduced sexual function with disease duration and pain [10]. The largest cohort of female SSc patients was analyzed in a multicenter study in 2012 by Levis et al. [3], who performed a multivariate logistic regression that assessed independent predictors of sexual activity/inactivity and sexual dysfunction. Out of the 547 women with SSc, 237 had complete data for all variables. Among the 165 sexually active patients, 62% reported impaired sexual function. Independent predictors of sexual impairment included older age, higher skin score, and more severe dyspnea. However, this study did not include HC and provided only descriptive statistics on rates of sexual activity and impairment [3]. 

A recent study by Gigante et al. [11] investigated the role of endothelial growth factor and endostatin in the pathogenesis of female sexual dysfunction in SSc patients. The authors demonstrated that reduced clitoral blood flow was caused by macro- and microvascular damage and impaired angiogenesis. Furthermore, Nazarinia et al. [12] showed significantly lower FSFI scores in 80 women with SSc compared to 80 HC adjusted for age. No significant association was found between vascular complications and sexual impairment among SSc patients. However, these results may have been influenced by specific cultural and religious factors since the study was conducted in Iran [12]. 

To our knowledge, only three studies have reported on the sexual health of SSc patients relative to HC. However, the study by Nazarinia et al. [12] is derived from a culturally specific population, and the studies of Schouffoer et al. [9] and Rosato et al. [13] were limited by the small sample size. Thus, there is an unmet need to provide further evidence on the impact of SSc on sexual health. This cross-sectional study aimed to assess female sexual dysfunction and pelvic floor muscle function in a considerable cohort of SSc patients compared to HC matched by age, in order to offer a comprehensive evaluation of sexual impairment in women with SSc. We also aimed to uncover a wide range of potential impacts of disease-related features on the patients’ sexual health including disease duration, disease activity, the presence of SSc symptoms, the severity of SSc impairment, overall functional ability, physical activity, extent of depression, fatigue, current pharmacotherapy, and quality of life. Using multivariate logistic regression, we aimed to assess independent predictors of sexual dysfunction in women with SSc. Furthermore, we conducted an additional analysis in sexually active patients as well as in patients of childbearing age, to circumvent bias due to lack of sexual activity or physiological changes after menopause. We also performed comparative analyses on patients with lcSSc and dcSSc as well as on patients with low and high disease activity. 

## 2. Methods

### 2.1. Patients and Healthy Controls

In total, we recruited 90 female patients (lcSSc/dcSSc: 61/29; mean ± SD age: 49.1 ± 11.6 years) consecutively at the Institute of Rheumatology in Prague from January 2018 to December 2020. The inclusion criteria included the fulfillment of the EULAR (European League Against Rheumatism)/ACR (American College of Rheumatology) classification criteria for SSc in 2013 [14] and patients between the ages of 18–70. The exclusion criteria was comprised of severe chronic comorbid diseases (further specified in the online Supplementary Materials) and any other systemic rheumatic disease. Regular follow-up examinations were performed by a rheumatologist and signed written informed consent was obtained from all participants. Ninety healthy subjects were recruited from the Healthy Control Register at the Institute of Rheumatology using the snowball method. These subjects were matched by age and consisted predominantly of employees and their relatives who do not have rheumatic diseases or severe chronic illnesses. This study was approved by the Ethics Committee at the Institute of Rheumatology in Prague. All procedures were conducted according to the relevant regulations and guidelines. 

### 2.2. Assessment Methods

All patients were evaluated by experienced rheumatologists (MT, RB), filled in 13 questionnaires that were well-established and widely validated, and received routine laboratory examinations. We collected the following data:

1. Demographic characteristics. Age at recruitment, education levels (primary, secondary, higher education), current sexual partnership, body mass index (BMI), alcohol intake, and smoking conditions.
2. Clinical features. Disease duration (from the first SSc symptom except for Raynaud’s phenomenon), disease activity determined by the European Scleroderma Study Group (ESSG) SSc activity score [15], involvement of the skin evaluated by the modified Rodnan skin score (mRSS) [16], and current medical therapy. Capillaroscopy and pulmonary function tests were routinely performed using standard methods [17,18]. All assessments were performed according to well-established guidelines [19], and all clinical features were documented.
3. Patient-reported outcomes (PROs). Fatigue was assessed by the Multidimensional Assessment of Fatigue Scale (MAF) [20] and the Fatigue Impact Scale (FIS) [21]. Depression was evaluated by the Beck’s Depression Inventory-II (BDI II) [22]. We used the Human Activity Profile (HAP) [23] to assess physical activity, and the Scleroderma Health Assessment Questionnaire (SHAQ) [24] and the Health Assessment Questionnaire (HAQ) [25] for functional status. The overall quality of life was analyzed using the 36-Item Short Form Survey (SF-36) [26]. Detailed descriptions have been stated elsewhere [27]. The Czech version of all questionnaires has previously been validated [28,29,30,31,32]. The importance of sexual life was subjectively assessed and recorded by a visual analog scale (VAS) ranging from 0 (not important at all) to 10 (extremely important).
4. Laboratory evaluation. Serum concentrations of C-reactive protein (CRP), erythrocyte sedimentation rate (ESR), antinuclear antibodies (ANA), and autoantibodies of the ENA complex were analyzed as described elsewhere [33].
5. Gynecological features. Previous pelvic surgery, menstrual status, hormone replacement therapy, contraception use, reasons for lack of sexual activity, and disease-related symptoms affecting sexual activity were recorded.
6. Sexual function evaluation (PROs). Sexual function in women was assessed by the Female Sexual Function Index (FSFI) [34], the Brief Index of Sexual Functioning for Women (BISF-W) [35], and the Sexual Function Questionnaire (SFQ-28) [36]. The impact of female sexual dysfunction on the quality of life was assessed by the Sexual Quality of Life Questionnaire-Female (SQoL-F) [37]. The Czech version of all questionnaires has been validated [38]. Further details are provided elsewhere [27].
7. Pelvic floor function evaluation (PROs). The sexual performance of women with pelvic floor problems was assessed using the Pelvic Organ Prolapse/Urinary Incontinence Sexual Questionnaire Short Form (PISQ-12) [39]. We also used Pelvic Floor Impact Questionnaire-Short Form 7 (PFIQ-7) [40] to determine the impact of pelvic floor dysfunction on the patients’ quality of life. Both questionnaires were translated into Czech and have been validated [38]. Further details are provided elsewhere [27].

### 2.3. Statistical Analysis

Data were first checked for normality by Shapiro–Wilk and Kolmogorov–Smirnov tests, and is expressed as mean ± standard deviation (SD) or median [interquartile range (IQR)] accordingly. Comparison between the two groups (SSc patients and HC) was conducted by the Mann–Whitney U test or the independent sample T-test. Differences between categorical variables were evaluated by the Chi-squared test. The bivariate relationships between variables pertaining to sexual function and clinical features were investigated using the point-biserial correlation and the Spearman correlation coefficient based on the variable type. For predictive analysis, we applied multiple linear regression to predict patients’ PRO scores for pelvic floor function and sexual function based on a set of predictors. Predictors of dependent variables were selected based on the significant associations in the bivariate analysis and clinical considerations. In case of multicollinearity, we selected only one predictor with the strongest correlation with the particular dependent variable to be included in the regression analysis. We also conducted a regression analysis for predictors selected based on clinical relevance and available literature, regardless of whether they were significantly associated with the dependent variable in the bivariate analysis. Statistical significance was defined as *p*-values of less than 0.05. All analyses were performed using SPSS version 25 (SPSS, Inc., Chicago, IL, USA) and GraphPad Prism 5 (version 5.02; GraphPad Software, La Jolla, CA, USA).

## 3. Results 

Among the 154 women with SSc initially recruited, 90 patients completed the questionnaires. Fifty-one subjects dropped out of the study for the following reasons: eight (16%) refused to participate in any research, 29 (57%) declined to answer sensitive questions, and 14 (27%) were uninterested or unconcerned. Thirteen patients were excluded due to missing data. Baseline demographic, clinical and laboratory data, and pharmacological treatment of the 90 analyzed patients and HC are listed in Table 1.

**Figure 1:**
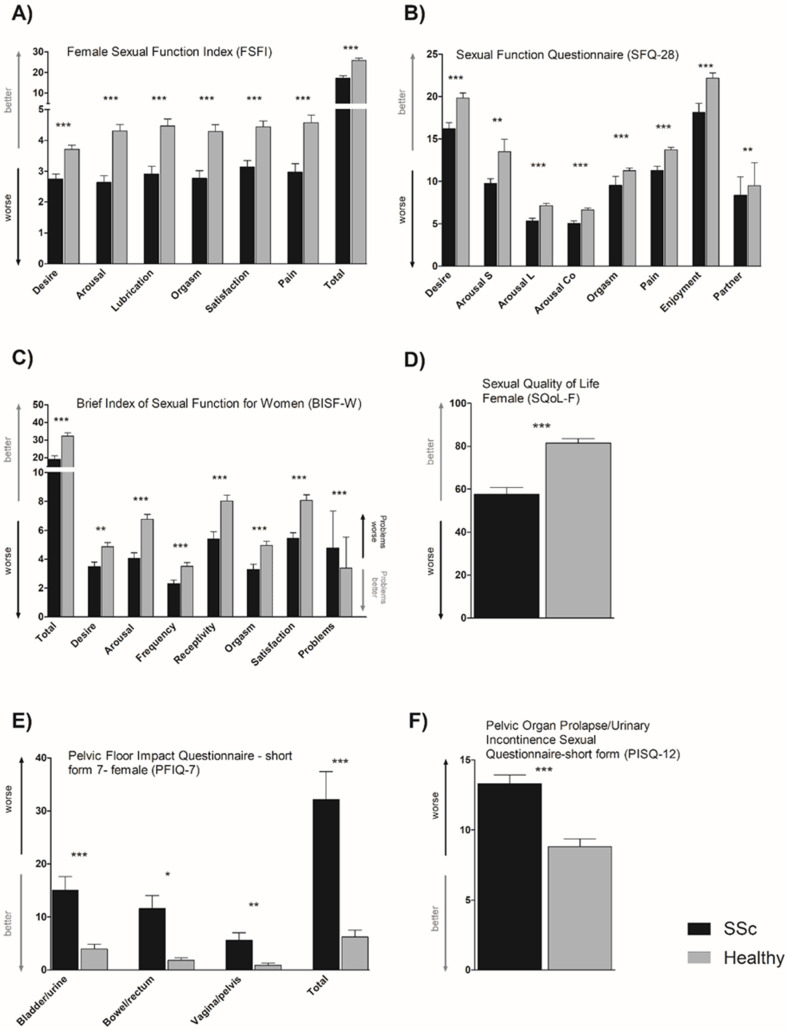
Female sexual function and pelvic floor muscle function in women with systemic sclerosis compared to healthy controls. (**A**) FSFI suggested impaired sexual function. (**B**) SFQ-28 indicated worse scores in all subscales in SSc patients, including desire, arousal lubrication (L), arousal sensation (S), arousal cognitive (Co), orgasm, enjoyment, pain, and partner. (**C**) BISF-W revealed worse scores in SSc patients for both total and subdomains of arousal, desire, receptivity/initiation, frequency of sexual activity, satisfaction, orgasm, and problems affecting sexual function. (**D**) Sexual quality of life (SQoL-F) was considerably undermined in SSc patients. Pelvic floor muscle function was impaired in SSc patients, as evidenced by both the PFIQ-7 (total score and all its subdomains) (**E**) and PISQ-12 (**F**) questionnaires. The complete title of the questionnaires is specified in the headings of the graphs. Data are plotted as mean (columns) with standard error of the mean (whiskers). *, *p* < 0.05; **, *p* < 0.01 ***, *p* < 0.001.

Compared to HC, patients with SSc reported significantly lower scores in all three questionnaires assessing sexual function (FSFI, BISF-W, and SFQ28), both in total scores (if applicable) and in all domains. Similarly, the quality of sexual life was significantly reduced in patients with SSc compared to HC (Figure 1 and Supplementary Table S1). Based on the FSFI cutoff score, the prevalence of female sexual dysfunction was 73% in SSc patients (vs. 31% in HC). Analyses on PFIQ-7 as well as PISQ-12 indicate impaired pelvic floor function compared to HC. According to PFIQ-7, bladder, rectum, and genital function were significantly affected (Figure 1 and Supplementary Table S1). Out of 90 SSc patients, 28 had not been sexually active in the previous four weeks due to systemic sclerosis (n = 10), health status of the partner (n = 4), absence of a sexual partner (n = 9), partnership difficulties (n = 1), and personal decision (n = 4). Out of the 62 sexually active patients, 37 (60%) reported that SSc-related symptoms limited their sexual activity and these symptoms were: restricted range of movement (n = 12), arthralgia (n = 11), decreased libido (n = 10), dyspareunia (n = 9), fatigue (n = 7), insufficient lubrication (n = 5), Raynaud’s phenomenon and hands’ contractures (n = 5), vaginal tightness (n = 5), body image dissatisfaction (n = 3), and dyspnea (n = 2).

According to our bivariate analysis, worse sexual performance and pelvic floor dysfunction significantly correlated with increased systemic inflammation and higher disease activity as well as the presence of dyspnea and interstitial lung disease. They also correlated with worse functional disability (HAQ), SSc-related impairment (SHAQ-Global), more pronounced fatigue, reduced physical activity, more severe depression, the use of antidepressants, and impaired overall quality of life. Surprisingly, higher levels of education and alcohol consumption were associated with better sexual function in patients with SSc (Table 2 and Supplementary Table S2). According to multivariate linear regression (Table 3), disease activity, SSc-related impairment, lung involvement, and fatigue were independently associated with decreased sexual function and quality of sexual life. Furthermore, SSc-related impairment and obstipation were demonstrated to be reliable predictors of pelvic floor muscle dysfunction in SSc patients. The results of multivariate regression with predictors selected based on clinical relevance and available literature are presented in Supplementary Table S3. 

Additionally, we noticed a significantly different number of sexually active individuals between the groups. Therefore, we compared the results between the 62 sexually active SSc patients (mean age ± SD: 46.9 ± 10.4 years) and 80 sexually active healthy controls (mean age ± SD: 47.9 ± 11.4 years). In this analysis, all the aforementioned differences remained significant, except for the bowel/rectum domain of PFIQ-7 (Table 4). 

Furthermore, we exclusively analyzed women of reproductive age since 50 patients and 49 healthy individuals were postmenopausal women. In this additional sub-analysis, we included 40 SSc patients (mean age ± SD 39.2 ± 7.5 years) and 41 healthy controls (mean age ± SD 40.1 ± 8.6 years). This subanalysis rendered similar results, and we observed significantly worse scores in premenopausal SSc women in all questionnaires and their subscales, except for the bowel/rectum domain of PFIQ-7 (Table 5). 

In addition, when we compared patients with lcSSc (n = 61, mean age ± SD 49.2 ± 11.2 years) to dcSSc (n = 29, mean age ± SD 49.0 ± 12.6 years), we did not detect any statistically significant differences in terms of sexual function and pelvic floor function. However, patients with higher disease activity (ESSG > 3, n = 22, mean age ± SD 50.5 ± 11.6 years) exhibited significantly worse scores in several indexes of sexual function than those with lower disease activity (ESSG ≤ 3, n = 68, mean age ± SD 48.7 ± 11.7 years) (Supplementary Table S4). 

## 4. Discussion

In this study, we demonstrated that women with SSc exhibited significantly worse pelvic floor function and sexual function than HC matched by age. Furthermore, worse assessment scores in these patients were associated with increased systemic inflammation, higher disease activity, several disease-related physical and psychological features, and impaired overall quality of life. According to multivariate regression, disease activity, SSc-related impairment, and lung involvement might be reliable predictors of female sexual dysfunction in SSc patients. 

Our findings are congruent with previous studies on sexual dysfunction in women with SSc. A similar study by Nazarinia et al. [12] demonstrated significantly lower total FSFI scores and their subscales other than the lubrication and pain domains in the patient group. However, the cultural and religious disparity makes it difficult to directly compare this Iranian study with ours. The mean FSFI scores of the healthy Iranian group [12] were markedly lower than those of our HC or of a Dutch population [9], especially in the domains of pain (mean ± SD: 2.4 ± 1.3 [12]/4.6 ± 2.3/5.0 ± 1.8 [9]) and lubrication (mean ± SD: 2.7 ± 1.2 [12]/4.7 ± 2.2/5.3 ± 1.1 [9]). Moreover, only married women were included in the Iranian study. Sexually active unmarried women were excluded due to social taboos. Therefore, the results cannot be fully generalized to the non-Islamic population. Consistent with our findings, impaired lubrication has been previously found to be common among women with SSc [2,41], especially in those with Sjögren’s syndrome [4]. Consequently, insufficient lubrication leads to uncomfortable or painful sexual intercourse. Therefore, there is no longer any doubt that women with SSc are more likely to have vaginal mucosal dryness and pain during sexual intercourse. These findings could raise the awareness of rheumatologists on these issues and include the use of lubricating gels in routine SSc recommendations and regimens.

Furthermore, FSFI was used to assess sexual performance in SSc patients in two additional studies [3,6]. The mean total score of FSFI (24.9 ± 6.7) in a sexually active SSc population analyzed by Impens et al. [6] was comparable to our results of sexually active patients (22.4 ± 9.2). Nevertheless, the other studies used either the 9-item FSFI version [3], a different screening tool [8,10], or the questionnaires lacked sufficient validation [4,7]. Hence, no other comparison can be provided. According to the FSFI cutoff score, sexual dysfunction was observed in 73% of SSc patients (vs. 31% in HC). Similarly, in the study by Schouffoer et al. [9] and Levis et al. [3], the reported prevalence was 70% and 62%, respectively. Therefore, we can conclude that approximately two thirds of SSc women might experience some degree of sexual dysfunction.

Admittedly, it might be challenging to describe the exact impact of SSc on sexual health considering the multifactorial etiologies of sexual functioning with biopsychological and socioenvironmental components, patient individuality, and the chronic nature of the disease. Nevertheless, we tried to investigate all factors that could be linked to sexual dysfunction in female patients with SSc and performed bivariate and multivariate analyses. In the bivariate analysis, worse sexual function was significantly associated with higher disease activity, dyspnea, worse SSc-related impairment, functional disability, more pronounced fatigue, reduced physical activity, antidepressant usage, more severe depression, and decreased overall quality of life. However, there is a general presumption that sexual health, fitness level, overall quality of life, fatigue, and depression interact with each other. In our study, we also observed that these variables are associated with several domains of sexual function in the healthy population. Nevertheless, these correlations were less frequent and were considerably weaker compared to the correlations in the SSc cohort (data not shown). From this, we can infer that the associations with these general variables can be even more pronounced in patients with SSc.

Interestingly, alcohol consumption and a higher education level were linked to better sexual function. There is some agreement with previous studies addressing this topic, although some findings may be contradictory. For example, a multicenter study by Levis et al. [3] suggested that patients with dyspnea and higher skin scores are more likely to be sexually impaired and patients who consume alcohol are less likely to be sexually active. In our study, the mRSS correlated to none of the sexual function indexes. To our knowledge, no other study has investigated this particular association. However, we assume that stiffening of the skin, leading to finger flexion contracture, limited range of movement, and microstomia, can negatively impact sexual foreplay, masturbation, and difficulty to assume some sex positions. On the other hand, a small amount of alcohol can have a positive effect on sexual activity as it can make the atmosphere more relaxed and relieve concerns about the appearance and body image dissatisfaction. In our cohort of women with SSc, the average weekly alcohol consumption (wine/beer) was 1.5 deciliters per week.

Consistent with previous results by Nazarinia et al. [12], no correlations were found between vascular involvement (manifested as Raynaud’s phenomenon, digital ulcers, pulmonary hypertension, renal crisis, and capillaroscopy findings) and sexual impairment among women with SSc. What remains unclear is the association between longer disease duration and sexual dysfunction. In this study, no correlation was observed, while in the study by Knafo et al. [10], this association was confirmed in both univariate and multivariate analyses. In line with our findings, three other studies [3,6,9] did not find any significant correlation between the disease duration and sexual dysfunction in women with SSc. On the other hand, consistent with our findings was the significant association of impaired sexual function with more severe depression and the use of antidepressants according to Schoulffoer et al. [9], and a more extensive deterioration of quality of life [6]. 

Generally, dcSSc is associated with more severe clinical manifestations and a worse prognosis than lcSSc [42]. Therefore, we would hypothesize a lower sexual function in patients with dcSSc. However, we did not observe any differences between patients with lcSSc and dcSSc. The only study analyzing these disease subtypes is the Iranian study by Nazarinia et al. [12], which showed significantly worse FSFI scores (total and three subscale scores) in 46 women with dcSSc than in 34 women with lcSSc. Nevertheless, since the numbers of lcSSc and dcSSc patients in our study were highly unbalanced, these results need to be interpreted with caution. It is to be expected that patients with more severe disabilities and a worse prognosis may have decreased sexual activity.

Even though this is the most comprehensive inquiry into sexual function in women with SSc, our study has several limitations. First, the investigation of sexual function can be cumbersome with reporting bias, particularly due to its inherent complexity, individual perception, and subjective evaluation using PROs. To examine sexual health in the broadest possible context, we investigated many factors that could potentially affect sexual function and used multiple screening tools. However, it needs to be emphasized that all correlations were weak to moderate, and the confidence intervals of the regression coefficients were large. Therefore, these results need to be interpreted with caution and always be considered at the individual level of each patient. 

Admittedly, the associations we have established based on the bivariate and multivariate correlations do not provide evidence of causality between selected clinical parameters and sexual dysfunction/pelvic floor dysfunction. In addition, there are several aspects we did not investigate such as the partner’s sexual function, the duration of the relationship, and history of sexual abuse. Multiple screening tools could allow for increased significant results by p-hacking. Nonetheless, the total scores and all domains of all three questionnaires evaluating sexual function rendered the same results. Indeed, we verified and validated these differences and provided further information on the unique subdomains. However, it is usually not possible to perform all questionnaires in clinical practice. In such cases, we would recommend using the FSFI since it is the most widely accepted questionnaire for sexual health including an extensive survey of the psychometric characteristics and is easy to complete [43].

Moreover, since our HC consisted predominantly of healthy employees of the institute and their relatives, the Healthy Worker Effect (HWE) [44] could have occurred in this study. Most notably, the subjects were from a single center and the sample size of 90 was not large enough to render robust and universally valid evidence. Therefore, these results need to be validated by multicenter, large-sampled research collaborations. 

## 5. Conclusions

To conclude, we demonstrated significantly more impaired sexual function and pelvic floor function in women with SSc compared to age-matched HC. In addition, bivariate analysis demonstrated that worse scores in SSc patients were associated with higher disease activity, increased systemic inflammation, the presence of interstitial lung disease and dyspnea, worse SSc-related impairment, functional disability, more pronounced fatigue, reduced physical activity, antidepressant utilization, more severe depression, and impaired overall quality of life. Moreover, the multivariate regression analysis confirmed that disease activity, SSc-related impairment, and lung involvement were reliable predictors of sexual dysfunction in SSc women. No differences between patients with lcSSc and dcSSc were observed. This study provides a comprehensive evaluation of female sexual health in SSc, bringing several new insights into this often neglected issue including pelvic floor function that has not yet been described in SSc, and will hopefully help facilitate holistic care for SSc patients. Despite the limitation of a relatively small sample size, this study should prompt future larger and multicentric studies to verify these results.

## Figures and Tables

**Table 1:** Sociodemographic characteristics, disease-related laboratory, and clinical features of SSc patients and healthy controls.

Parameters	SSc (n = 90)	Healthy Controls (n = 90)	*p*
**Sociodemographic variables**			
Age, years	49.5 (38.8–58.8)	49.5 (38.8–58.8)	0.9999
Having a partner, n (%)	70 (78)	82 (91)	**0.0223**
Education level (primary/secondary/tertiary), n (%)	1 (1)/60 (67)/29 (32)	0 (0)/58 (64)/32 (36)	0.5349
**Sexual health features**			
Sexual activity, n (%)	62 (69)	80 (89)	**0.0019**
Menopause, n (%)	50 (56)	49 (54)	0.9997
Pelvic surgery, n (%)	16 (18)	7 (8)	0.0675
Normal menstrual cycle, n (%)	22 (55)	-	
Contraceptives, n (%)	5 (13)	-	
HRT, n (%)	6 (12)	-	
VAS: sexual life importance	6.5 (3.8–8.0)	7.0 (5.0–8.3)	0.0649
**Clinical features**			
Disease duration, years	4.0 (2.0–8.0)	-	
SSc subtype: lcSSc/dcSSc, n (%)	61 (68)/29 (32)	-	
ESSG activity index	2.0 (1.0–3.1)	-	
mRSS	5.0 (0.0–13.0)	-	
SSc-associated symptoms: n (%)		-	
ILD/PAH/OD/P/RI	51 (57)/13 (15)/56 (63)/4 (5)/33 (39)	-	
RP/DU/CA/A/MW	83 (92)/41 (46)/8 (9)/46 (52)/13 (15)	-	
**Patient-reported outcomes**			
(**score range worst–best)**			
FIS: fatigue (160–0)	57.5 (33.5–86.3)	26.0 (7.0–46.8)	**0.0002**
MAF: fatigue (50–1)	27.8 (19.4–35.7)	14.4 (9.1–21.1)	**0.0002**
BDI-II: depression (63–0)	14.0 (7.0–20.0)	4.5 (1.0–8.0)	**0.0002**
HAP AAS: physical activity (0–94)	54.0 (35.5–72.5)	82.0 (76.0–91.5)	**0.0002**
HAQ: functional status (3–0)	0.75 (0.2–1.4)	0.0 (0.0–0.0)	**0.0002**
SHAQ: SSc-related functional status (3–0)	1.0 (0.5–1.4)	-	
Global SHAQ: SSc impairment (3–0)	0.9 (0.4–1.4)	-	
SF-36 PCS: quality of life (16.6–57.9)	31.1 (24.9–43.0)	53.9 (46.0–56.8)	**0.0002**
SF-36 MCS: quality of life (5.5–63.6)	42.6 (31.0–50.5)	52.4 (47.2–57.0)	**0.0002**
**Laboratory features**			
Autoantibodies, seronegative, n (%):	3 (3)	-	
ANA/Scl70/ACA/RNA3P n (%)	86 (96)/45(50)/20(22)/2 (2)	-	
CRP, mg/L	3.2 (1.6–6.5)	-	
ESR, mm/h	12.0 (8.0–25.0)	-	
**Current treatment**			
Prednisone equivalent dose, mg/day	0.0 (0.0–2.5)	-	
GC/MTX/CPA/AZA/MMF, n (%)	26 (29)/20 (22)/19 (21)/9 (10)/8 (9)	-	
RTX/TCZ/CCB/antiHT/bosentan, n (%)	3 (3)/1 (1)/4 (4)/15 (17)/6 (7)	-	
sildenafil/epoprostenol/alprostadil, n (%)	4 (4)/1 (1)/12 (13)	-	
antidepressants and mood stabilizers, n (%)	18 (20)	-	

Notes: Data are presented as median (IQR), unless stated otherwise. Statistically significant differences (*p* < 0.05) are highlighted in bold; IQR, interquartile range; SSc, systemic sclerosis; lcSSc, limited cutaneous SSc; dcSSc, diffuse cutaneous SSc; HRT, hormone replacement therapy; VAS, visual analog scale; ESSG, European Scleroderma Study Group; mRSS, modified Rodnan skin score; ILD, interstitial lung disease; PAH, pulmonary arterial hypertension; OD, esophageal dysmotility; P, palpitation; RI, renal involvement; RP, Raynaud’s phenomenon; DU, digital ulceration; CA, calcification; A, arthritis; MW, muscle weakness; Scl-70, anti–DNA-topoisomerase I antibodies; ANA, antinuclear antibodies; ACA, anticentromere antibodies; RNA3P, RNA polymerase III antibodies; CRP, C-reactive protein; ESR, erythrocyte sedimentation rate; GC, glucocorticoids; MTX, methotrexate; CPA, cyclophosphamide; AZA, azathioprine; MMF, mycophenolate mofetil; RTX, rituximab; TCZ, tocilizumab; CCB, calcium channel blockers; antiHT, antihypertensives; MAF, Multidimensional Assessment of Fatigue; FIS, Fatigue Impact Scale; HAP AAS, Human Activity Profile Adjusted Activity Score; BDI II, Beck’s Depression Inventory-II; SF-36 MCS, Medical outcomes study Short Form 36-Mental Component Summary; SF-36 PCS, Medical outcomes study Short Form 36-Physical Component Summary; HAQ, Health Assessment Questionnaire; SHAQ, Scleroderma Health Assessment Questionnaire—a total score of five visual analog scales; Global SHAQ, aggregated score of HAQ and SHAQ.

**Table 2:** Spearman’s and Pearson’s * correlation coefficients of sexual function and pelvic floor function with disease-related laboratory and clinical features of SSc patients.

		FSFI Total	FSFI Desire	FSFI Arousal	FSFI Lubrication	FSFI Orgasm	FSFI Satisfaction	FSFI Pain	BISF-W Total	SQoL-F	PISQ-12	PFIQ-7 Total
ESSG	r	**−0.237**	−0.081	**−0.309**	**−0.254**	−0.178	**−0.268**	**−0.243**	**−0.261**	**−0.369**	**0.308**	0.028
	p	**0.025**	0.452	**0.003**	**0.016**	0.096	**0.011**	**0.022**	**0.015**	**0.001**	**0.005**	0.790
	n	**89**	89	**89**	**89**	89	**89**	**89**	**87**	**83**	**83**	90
ESR	r	−0.147	−0.140	**−0.239**	**−0.222**	−0.085	−0.087	−0.003	**−0.273**	−0.061	0.097	−0.171
	p	0.177	0.198	**0.027**	**0.040**	0.439	0.426	0.976	**0.012**	0.592	0.392	0.113
	n	86	86	**86**	**86**	86	86	86	**84**	80	80	87
Dyspnea	r	−0.227 *	−0.249 *	−0.246 *	−0.222 *	−0.250 *	−0.121 *	−0.134 *	−0.288 *	−0.106 *	0.168 *	0.168 *
	p	**0.034**	**0.019**	**0.021**	**0.037**	**0.019**	0.263	0.312	**0.007**	0.344	0.131	0.115
	n	**88**	**88**	**88**	**88**	**88**	88	88	**86**	82	82	89
ILD	r	−0.252 *	−0.329 *	−0.285 *	−0.264 *	−0.249 *	−0.153 *	−0.103 *	−0.347 *	−0.228 *	0.190 *	0.234 *
	p	**0.019**	**0.002**	**0.008**	**0.014**	**0.020**	0.157	0.342	**0.001**	**0.041**	0.089	**0.028**
	n	**87**	**87**	**87**	**87**	**87**	87	87	**85**	**81**	81	**88**
DLCO	r	**0.349**	0.217	**0.444**	**0.399**	**0.343**	**0.295**	**0.251**	**0.362**	**0.237**	**−0.208**	**−0.280**
	p	**0.001**	0.052	**0.001**	**0.001**	**0.002**	**0.008**	**0.024**	**0.001**	**0.039**	**0.074**	**0.011**
	n	**81**	81	**81**	**81**	**81**	**81**	**81**	**80**	**76**	**75**	**82**
Dysphagia/ pyrosis	r	**−0.215**	0.158	**−0.250**	**−0.211**	−0.202	−0.105	−0.200	−0.157	−0.104	0.135	0.177
	p	**0.044**	0.140	**0.019**	**0.049**	0.059	0.330	0.062	0.149	0.351	0.228	0.097
	n	**88**	88	**88**	**88**	88	88	88	86	82	82	89
Obstipation/ diarrhea	r	−0.184	**−0.273**	−0.180	−0.210	−0.200	−0.099	−0.067	**−0.220**	−0.093	**0.325**	0.163
	p	0.087	**0.011**	0.095	0.051	0.063	0.361	0.537	**0.043**	0.410	**0.003**	0.130
	n	87	**87**	87	87	87	87	87	**85**	81	**81**	88
Antidepressants	r	−0.343 *	−0.269 *	−0.339 *	−0.267 *	−0.378 *	−0.345 *	−0.220 *	−0.326 *	−0.383 *	0.336 *	0.076 *
	p	**0.002**	**0.017**	**0.002**	**0.017**	**0.001**	**0.002**	**0.050**	**0.004**	**0.001**	**0.003**	0.503
	n	**79**	**79**	**79**	**79**	**79**	**79**	**79**	**77**	**75**	**75**	80
SSc limitations	r	−0.464 *	−0.472 *	−0.337 *	−0.380 *	−0.270 *	−0.396 *	−0.435 *	−0.517 *	−0.487 *	0.437 *	−0.072 *
	p	**0.001**	**0.001**	**0.011**	**0.004**	**0.045**	**0.003**	**0.001**	**0.001**	**0.001**	**0.001**	0.947
	n	**53**	**53**	**53**	**53**	**53**	**53**	**53**	**53**	**53**	**55**	56
Education	r	0.241 *	0.228 *	0.284 *	0.223 *	0.164 *	0.260 *	0.159 *	0.270 *	0.295 *	−0.202 *	−0.146 *
	p	**0.023**	**0.033**	**0.007**	**0.037**	0.127	**0.014**	0.139	**0.012**	**0.007**	0.069	0.173
	n	**88**	**88**	**88**	**88**	88	**88**	88	**86**	**82**	82	82
Alcohol	r	**0.381**	**0.316**	**0.399**	**0.420**	**0.307**	**0.313**	**0.221**	**0.454**	**0.259**	−0.117	−0.185
	p	**0.001**	**0.005**	**0.001**	**0.001**	**0.006**	**0.005**	**0.050**	**0.001**	**0.025**	0.317	0.100
	n	**79**	**79**	**79**	**79**	**79**	**79**	**79**	**77**	**75**	75	80
SHAQ global	r	**−0.436**	**−0.392**	**−0.406**	**−0.353**	**−0.366**	**−0.366**	**−0.282**	**−0.303**	**−0.471**	**0.339**	**0.236**
	p	**0.001**	**0.001**	**0.001**	**0.002**	**0.001**	**0.001**	**0.013**	**0.008**	**0.001**	**0.004**	**0.038**
	n	**77**	**77**	**77**	**77**	**77**	**77**	**77**	**75**	**72**	**71**	**78**
HAQ	r	**−0.393**	**−0.354**	**−0.364**	**−0.307**	**−0.328**	**−0.344**	**−0.298**	**−0.280**	**−0.483**	**0.326**	0.144
	p	**0.001**	**0.001**	**0.001**	**0.004**	**0.002**	**0.001**	**0.005**	**0.009**	**0.001**	**0.003**	0.179
	n	**88**	**88**	**88**	**88**	**88**	**88**	**88**	**86**	**82**	**82**	89
BDI-II	r	**−0.506**	**−0.527**	**−0.455**	**−0.442**	**−0.416**	**−0.484**	**−0.328**	**−0.475**	**−0.532**	**0.385**	**0.451**
	p	**0.001**	**0.001**	**0.001**	**0.001**	**0.001**	**0.001**	**0.002**	**0.001**	**0.001**	**0.001**	**0.001**
	n	**88**	**88**	**88**	**88**	**88**	**88**	**88**	**86**	**82**	**82**	**89**
FIS	r	**−0.496**	**−0.484**	**−0.465**	**−0.398**	**−0.416**	**−0.461**	**−0.278**	**−0.477**	**−0.433**	**0.296**	**0.297**
	p	**0.001**	**0.001**	**0.001**	**0.001**	**0.001**	**0.001**	**0.008**	**0.001**	**0.001**	**0.007**	**0.005**
	n	**89**	**89**	**89**	**89**	**89**	**89**	**89**	**87**	**83**	**83**	**80**
HAP-AAS	r	**0.535**	**0.478**	**0.499**	**0.427**	**0.433**	**0.440**	**0.448**	**0.533**	**0.389**	**−0.325**	**−0.217**
	p	**0.001**	**0.001**	**0.001**	**0.001**	**0.001**	**0.001**	**0.001**	**0.001**	**0.001**	**0.003**	**0.041**
	n	**88**	**88**	**88**	**88**	**88**	**88**	**88**	**86**	**82**	**82**	**89**
SF-36 PCS	r	**0.382**	**0.412**	**0.355**	**0.308**	**0.289**	**0.301**	**0.244**	**0.332**	**0.388**	**−0.336**	−0.178
	p	**0.001**	**0.001**	**0.001**	**0.004**	**0.007**	**0.005**	**0.023**	**0.002**	**0.001**	**0.002**	0.098
	n	**87**	**87**	**87**	**87**	**87**	**87**	**87**	**85**	**81**	**81**	88
SF-36 MCS	r	**0.394**	**0.370**	**0.370**	**0.332**	**0.344**	**0.429**	**0.247**	**0.321**	**0.352**	**−0.266**	**−0.372**
	p	**0.001**	**0.001**	**0.001**	**0.002**	**0.001**	**0.001**	**0.021**	**0.003**	**0.001**	**0.017**	**0.001**
	n	**87**	**87**	**87**	**87**	**87**	**87**	**87**	**85**	**81**	**81**	**88**

Notes: Statistically significant correlations (*p* < 0.05) are marked in bold. Pearson’s correlation coefficients are marked with *. SSc, systemic sclerosis; ESSG, European Scleroderma Study Group; ESR, erythrocyte sedimentation rate; ILD, interstitial lung disease; DLCO, diffusing capacity of the lungs for carbon monoxide; SSc limitations, the presence of difficulties associated with systemic sclerosis limiting sexual activity; HAQ, Health Assessment Questionnaire; SHAQ Global, Scleroderma Health Assessment Questionnaire-Global Score, aggregated score of HAQ and SHAQ; HAP AAS, Human Activity Profile Adjusted Activity Score; FIS, Fatigue Impact Scale; BDI-II, Beck’s Depression Inventory-II; SF-36 MCS, Medical outcomes study Short Form 36-Mental Component Summary; SF-36 PCS, Medical outcomes study Short Form 36-Physical Component Summary; FSFI, Female Sexual Function Index; BISF-W, Brief Index of Sexual Function for Women; SQoL-F, Sexual Quality of Life-Female; PFIQ-7, Pelvic Floor Impact Questionnaire-short form 7; PISQ-12, Pelvic Organ Prolapse/Urinary Incontinence Sexual Questionnaire short form.

**Table 3:** Multivariate regression analysis predicting sexual function and pelvic floor function in female patients with SSc based on clinical features.

	*β* (95% CI)	Stand. β	*p*	Adjusted R^2^	*p* *
**FSFI**					
Overall model				0.219	**0.0001**
ESSG activity index	−1.281 (−2.966; 0.403)	−0.172	0.134		
DLCO	0.076 (−0.044; 0.196)	0.144	0.210		
SHAQ-Global	−8.062 (−12.557; 3.568)	−0.390	**0.001**		
**BISF-W**					
Overall model				0.292	**0.0001**
ESSG activity index	−2.129 (−4.632; 0.375)	−0.170	0.094		
ESR	−0.108 (−0.282; −0.066)	−0.123	0.221		
ILD	−7.195 (−14.319; −0.070)	−0.196	**0.048**		
FIS total	0.218 (−0.318; −0.118)	−0.412	**0.0001**		
**SQoL-F**					
Overall model				0.341	**0.0001**
ESSG activity index	−5223 (−9.211; −1.234)	−0.277	**0.011**		
DLCO	−0.045 (−0.328; 0.237)	−0.034	0.749		
SHAQ-Global	−25.974 (−36.470; 15.477)	−0.514	**0.0001**		
**PISQ-12**					
Overall model				0.207	**0.002**
ESSG activity index	0.868 (−0.006; 1.741)	0.223	0.052		
Obstipation/diarrhea	3.756 (0.716; 6.795)	0.277	**0.016**		
SHAQ-Global	2.232 (0.035; 4.429)	0.230	**0.047**		
**PFIQ-7**					
Overall model				0.162	**0.008**
SHAQ	29.973 (8.543; 51.403)	0.337	**0.007**		
DLCO	−0.380 (−0.949; 0.188)	−0.159	0.186		
HAP AAS	−0.063 (−0.651; 0.526)	−0.027	0.832		

Notes: Statistical significance (*p* < 0.05) is marked in bold. β, regression beta coefficient; stand. β, standardized regression beta coefficient; CI, confidence interval; *p*, *p*-value of the predictor in the model; Adjusted R2, R-squared adjusted for the number of predictors in the model; *p* *, *p* for the overall model; SSc, systemic sclerosis; FSFI, Female Sexual Function Index; BISF-W, Brief Index of Sexual Function for Women; SQoL-F, Sexual Quality of Life-Female; PISQ-12, Pelvic Organ Prolapse/Urinary Incontinence Sexual Questionnaire short form; PFIQ-7, Pelvic Floor Impact Questionnaire-short form 7; ESSG, European Scleroderma Study Group; ESR, erythrocyte sedimentation rate; DLCO, diffusing capacity of the lungs for carbon monoxide; SHAQ Global, Scleroderma Health Assessment Questionnaire-Global Score; SHAQ, Scleroderma Health Assessment Questionnaire—a total score of five visual analog scales; FIS, Fatigue Impact Scale, HAP AAS, Human Activity Profile Adjusted Activity Score.

**Table 4:** Sexual function and pelvic floor function in sexually active women with SSc and healthy controls.

Parameters (Score Range Worst–Best)	SA SSc (n = 62)	SA HC (n = 80)	*p*-Value
**FSFI total (range 2–36)** FSFI desire (range 1.2–6) FSFI arousal (range 0–6) FSFI lubrication (range 0–6) FSFI orgasm (range 0–6) FSFI satisfaction (range 0.8–6) FSFI pain (range 0–6) **BISF-W total (range −16–75)** BISF-W thoughts/desire (range 0–12) BISF-W arousal (range 0–12) BISF-W frequency of sexual activity (range 0–12) BISF-W receptivity/initiation (range 0–15) BISF-W pleasure/orgasm (range 0–12) BISF-W relationship satisfaction (range 0–12) BISF-W problems affecting sexual function (range 16–0) SFQ28 desire (range 5–31) SFQ28 arousal sensation (range 4–20) SFQ28 arousal lubrication (range 2–10) SFQ28 arousal cognitive (range 2–10) SFQ28 orgasm (range 1–15) SFQ28 pain (range 2–15) SFQ28 enjoyment (range 6–30) SFQ28 partner (range 2–10) **SQoL-F (range 0–100)** **PISQ-12 (range 48–0)** **PFIQ-7 total (range 300–0), mean ± SD** PFIQ-7 bladder/urine (range 100–0) PFIQ-7 bowel/rectum (range 100–0) PFIQ-7 vagina/pelvis (range 100–0)	24.0 (16.7–30.2) 3.3 (1.7–4.2) 3.6 (2.3–5.1) 4.4 (2.7–5.7) 4.2 (2.4–5.2) 4.4 (2.0–5.6) 4.8 (2.8–6.0) 30.4 (13.0–38.4) 4.3 (2.3–6.4) 6.3 (4.0–7.8) 3.3 (1.3–5.0) 8.0 (5.0–10.0) 4.8 (2.0–7.0) 7.0 (5.0–10.0) 5.1 (3.5–7.0) 17.0 (12.0–20.0) 10.0 (8.0–13.0) 6.0 (4.0–7.0) 5.0 (4.0–6.0) 10.0 (6.0–12.0) 12.0 (10.0–15.0) 19.0 (14.0–24.0) 9.0 (7.5–10.0) 65.6 (40.3–92.5) 12.0 (9.0–16.5) 26.3 ± 46.5 12.5 ± 23.5 8.3 ± 19.1 5.5 ± 13.5	30.8 (26.8–33.1) 3.6 (3.6–4.8) 5.1 (4.5–5.7) 5.9 (4.8–6.0) 5.2 (4.1–6.0) 5.2 (4.0–6.0) 6.0 (5.2–6.0) 39.4 (30.3–46.5) 5.7 (3.9–7.0) 7.5 (6.3–9.8) 4.0 (2.4–5.4) 10.0 (7.0–11.0) 5.9 (4.4–7.5) 10.0 (7.0–11.0) 2.9 (1.8–5.4) 21.0 (17.0–23.0) 12.0 (9.0–15.0) 8.0 (6.0–9.0) 7.0 (5.0–8.0) 12.0 (10.0–13.0) 15.0 (13.0–15.0) 23.0 (20.0–25.0) 10.0 (9.0–10.0) 91.1 (73.9–96.9) 7.0 (5.0–11.0) 6.1 ± 11.6 3.9 ± 8.3 1.8 ± 4.8 0.9 ± 3.6	**0.0002** **0.0005** **0.0002** **0.0002** **0.0010** **0.0003** **0.0001** **0.0005** **0.0187** **0.0003** **0.0437** **0.0191** **0.0349** **0.0007** **0.0002** **0.0006** **0.0034** **0.0002** **0.0001** **0.0001** **0.0002** **0.0006** **0.0014** **0.0002** **0.0002** **0.0016** **0.0326** 0.2324 **0.0035**

Notes: Data are presented as median (IQR), if not stated otherwise. Statistically significant differences (*p* < 0.05) are marked in bold. The number of respondents to the SFQ-28 questionnaire was 57 for SSc women and 75 for healthy women; IQR, interquartile range; SD, standard deviation; SSc, systemic sclerosis; HC, healthy controls; BISF-W, Brief Index of Sexual Function for Women; FSFI, Female Sexual Function Index; SFQ-28; Sexual Function Questionnaire; PISQ-12, Pelvic Organ Prolapse/Urinary Incontinence Sexual Questionnaire Short Form; PFIQ-7, Pelvic Floor Impact Questionnaire-Short Form 7; SQoL-F, Sexual Quality of Life-Female.

**Table 5:** Sexual function and pelvic floor function in women of reproductive age with SSc and healthy controls in the bowel/rectum domain of PFIQ-7 (Table 5).

Parameters (Score Range Worst–Best)	SSc in Reproductive Age (n = 40)	HC in Reproductive Age (n = 41)	*p*-Value
**FSFI total (range 2–36)** FSFI desire (range 1.2–6) FSFI arousal (range 0–6) FSFI lubrication (range 0–6) FSFI orgasm (range 0–6) FSFI satisfaction (range 0.8–6) FSFI pain (range 0–6) **BISF-W total (range −16–75)** BISF-W thoughts/desire (range 0–12) BISF-W arousal (range 0–12) BISF-W frequency of sexual activity (range 0–12) BISF-W receptivity/initiation (range 0–15) BISF-W pleasure/orgasm (range 0–12) BISF-W relationship satisfaction (range 0–12) BISF-W problems affecting sexual function (range 16–0) SFQ28 desire (range 5–31) SFQ28 arousal sensation (range 4–20) SFQ28 arousal lubrication (range 2–10) SFQ28 arousal cognitive (range 2–10) SFQ28 orgasm (range 1–15) SFQ28 pain (range 2–15) SFQ28 enjoyment (range 6–30) SFQ28 partner (range 2–10) **SQoL-F (range 0–100)** **PISQ-12 (range 48–0)** **PFIQ-7 total (range 300–0), mean ± SD** PFIQ-7 bladder/urine (range 100–0) PFIQ-7 bowel/rectum (range 100–0) PFIQ-7 vagina/pelvis (range 100–0)	24.5 (13.3–31.5) 3.6 (2.0–4.2) 3.8 (1.5–5.0) 5.1 (2.4–6.0) 3.6 (1.4–5.2) 4.0 (1.7–5.6) 5.2 (2.0–6.0) 28.8 (9.2–39.9) 5.1 (2.7–7.1) 6.3 (2.8–8.1) 3.0 (0.9–5.3) 7.5 (4.0–10.3) 4.6 (1.0–7.3) 7.0 (4.0–10.0) 4.7 (3.5–7.0) 19.0 (12.3–21.0) 11.0 (8.3–14.0) 7.0 (4.0–8.0) 6.0 (4.0–7.0) 10.0 (6.0–12.0) 13.0 (10.3–15.0) 20.0 (14.0–25.0) 9.0 (7.3–10.0) 64.4 (33.3–93.6) 12.0 (8.3–16.0) 22.2 ± 42.7 8.6 ± 16.5 7.3 ± 18.0 6.3 ± 14.5	32.2 (29.0–34.6) 4.8 (3.6–5.4) 5.7 (4.8–5.7) 6.0 (5.6–6.0) 5.6 (4.6–6.0) 5.2 (4.2–6.0) 6.0 (6.0–6.0) 44.3 (35.0–48.0) 6.6 (5.2–7.6) 8.8 (7.0–10.0) 4.7 (3.1–6.0) 10.0 (8.0–11.0) 6.7 (5.0–7.8) 10.0 (8.3–11.0) 2.4 (1.5–4.8) 22.0 (19.5–24.0) 14.0 (11.0–17.0) 8.0 (7.0–9.0) 8.0 (6.5–8.5) 12.0 (10.0–13.0) 15.0 (15.0–15.0) 25.0 (22.0–26.5) 10.0 (10.0–10.0) 92.8 (78.6–96.7) 7.0 (4.0–10.0) 3.7 ± 8.1 2.6 ± 6.8 1.2 ± 3.0 0.0 ± 0.0	**0.0002** **0.0006** **0.0002** **0.0003** **0.0008** **0.0068** **0.0005** **0.0022** **0.0187** **0.0003** **0.0288** **0.0175** **0.0206** **0.0005** **0.0003** **0.0017** **0.0082** **0.0021** **0.0001** **0.0001** **0.0002** **0.0061** **0.0004** **0.0001** **0.0002** **0.0057** **0.0276** 0.5382 **0.0001**

Notes: Data are presented as median (IQR), unless stated otherwise. Statistically significant differences (*p* < 0.05) are highlighted in bold. Thirty-two SSc women and 37 healthy women completed the SFQ-28 questionnaire; IQR, interquartile range; SD, standard deviation; SSc, systemic sclerosis; HC, healthy controls; BISF-W, Brief Index of Sexual Function for Women; FSFI, Female Sexual Function Index; SQoL-F, Sexual Quality of Life-Female; SFQ-28, Sexual Function Questionnaire; PFIQ-7, Pelvic Floor Impact Questionnaire-Short Form; PISQ-12, Pelvic Organ Prolapse/Urinary Incontinence Sexual Questionnaire Short Form 7.

## Data Availability

Individual anonymized participant data will not be shared. Pooled study data, protocol, or statistical analysis plan can be shared upon request at hermanko-va@revma.cz.

## Supplementary Materials

The following supporting information can be downloaded at: https://www.mdpi.com/article/10.3390/ijerph19010612/s1, Table S1: Sexual function and pelvic floor function in women with SSc and healthy controls; Table S2: Spearman’s and Pearson’s* correlation coefficients of sexual function and pelvic floor function with disease-related laboratory and clinical features SSc patients; Table S3: Sexual function and pelvic floor function in SSc patients with low and high disease activity assessed by the European Scleroderma Study Group Activity Index; Table S4: Multivariate regression analysis predicting sexual function and pelvic floor function in female patients with SSc based on clinical features.
