# Supplementary material for: Female Sexual Dysfunction and Pelvic Floor Muscle Function Associated with Systemic Sclerosis: A Cross-Sectional Study
Source: Int J Environ Res Public Health. 2022 Jan 5;19(1):612. doi: 10.3390/ijerph19010612 (PMC8744868; doi:10.3390/ijerph19010612)
Supplement: Supplementary file 1 [file ijerph-19-00612-s001.zip › ijerph-1497781-supplementary.pdf]

Sexual Function and Pelvic Floor Function in Women with Systemic Sclerosis: A Cross-sectional Study

– supplementary material–

SUPPLEMENTARY METHODS

Exclusion criteria

Exclusion criteria for both groups (in SSc patients as a comorbid condition) comprised severe chronic diseases as defined by World Health Organization (WHO) (1), namely: active neoplasia (recent diagnosis of cancer with ongoing treatment), severe cardiovascular disease, chronic respiratory disease, severe neurological and mental disorder.

Scleroderma Health Assessment Questionnaire

The modified Scleroderma Health Assessment Questionnaire (SSc HAQ) has been validated in 1997 (2) and was proposed as a more specific version of the HAQ for SSc by adding five specific patient-reported visual analog scales (VAS) to assess gastrointestinal and pulmonary symptoms, Raynaud's phenomenon, digital ulcers, and overall disease severity. These five aspects are graded from 0 (no interference with patient's activity) to 3 (very severe limitation). The mean of these five scores has been calculated as the "SHAQ" score (2). An aggregated score called "Global SHAQ" ranging from 0 (no disability) to 3 (maximum disability) was calculated according to Georges et al. (3) as follows: SSc HAQ = (8 HAQ domains scores + 5 VAS scores) divided by 13.

SUPPLEMENTARY RESULTS

Supplementary Table S1. Sexual function and pelvic floor function in women with SSc and healthy controls

Parameters (score range worst-best)	SSc (n = 90)	Healthy Controls (n = 90)	p-value
**FSFI total (range 2-36)**	19.4 (3.9-26.8)	30.2 (23.1-32.9)	**p = 0.0002**
FSFI desire (range 1.2-6)	2.4 (1.2-3.6)	3.6 (3.0-4.8)	**p = 0.0002**
FSFI arousal (range 0-6)	2.7 (0.5-4.7)	5.1 (4.1-5.7)	***p* = 0.0002**
FSFI lubrication (range 0-6)	3.7 (0.0-5.3)	5.7 (3.6-6.0)	***p* = 0.0002**
FSFI orgasm (range 0-6)	3.2 (0.0-4.8)	5.2 (3.6-6.0)	***p* = 0.0002**
FSFI satisfaction (range 0.8-6)	3.2 (1.0-5.2)	5.2 (3.6-6.0)	***p*<0.0001**
FSFI pain (range 0-6)	3.6 (0.0-5.4)	6.0 (4.3-6.0)	***p* = 0.0002**
**BISF-W total (range -16-75)**	14.3 (2.1-35.1)	38.2 (19.3-46.2)	***p* = 0.0002**
BISF-W thoughts/desire (range 0-12)	3.3 (1.0-5.9)	5.3 (3.0-6.9)	***p* = 0.0012**
BISF-W arousal (range 0-12)	4.3 (0.3-7.3)	7.0 (5.3-9.3)	***p* = 0.0002**
BISF-W frequency of sexual activity (range 0-12)	1.7 (0.3-4.0)	3.6 (1.8-5.3)	***p* = 0.0006**
BISF-W receptivity/initiation (range 0-15)	5.0 (0.0-9.0)	10.0 (6.0-11.0)	***p* = 0.0002**
BISF-W pleasure/orgasm (range 0-12)	2.5 (0.0-5.8)	5.5 (3.3-7.3)	***p* = 0.0005**
BISF-W relationship satisfaction (range 0-12)	6.0 (2.0-9.0)	9.0 (6.0-11.0)	***p* = 0.0002**
BISF-W problems affecting sexual function (range 16-0)	4.4 (3.0-6.6)	2.7 (1.8-5.3)	***p*<0.0001**
SFQ-28 desire (range 5-31)	17.0 (12.0-20.0)	21.0 (17.0-23.0)	***p* = 0.0002**
SFQ-28 arousal sensation (range 4-20)	10.0 (8.0-13.0)	12.0 (9.0-15.0)	***p* = 0.0022**
SFQ-28 arousal lubrication (range 2-10)	5.0 (4.0-7.0)	8.0 (6.0-9.0)	***p* = 0.0002**
SFQ-28 arousal cognitive (range 2-10)	5.0 (4.0-6.0)	7.0 (5.0-8.0)	***p* = 0.0002**
SFQ-28 orgasm (range 1-15)	10.0 (6.0-12.0)	12.0 (10.0-13.0)	***p* = 0.0002**
SFQ-28 pain (range 2-15)	12.0 (9.5-15.0)	15.0 (13.0-15.0)	***p* = 0.0002**
SFQ-28 enjoyment (range 6-30)	19.0 (12.5-24.0)	23.0 (20.0-25.0)	***p*<0.0001**
SFQ-28 partner (range 2-10)	9.0 (8.0-10.0)	10.0 (9.0-10.0)	***p* = 0.0014**
**SQoL-F (range 0-100)**	61.1 (34.4-81.1)	91.1 (70.0-96.7)	***p*<0.0001**
**PISQ-12 (range 48-0)**	13.0 (9.0-17.0)	7.0 (5.0-12.0)	***p* = 0.0002**
**PFIQ-7 total (range 300-0), mean ± SD**	31.8 ± 48.3	6.2 ± 11.8	***p* = 0.0002**
PFIQ-7 bladder/urine (range 100-0)	14.6 ± 23.7	3.9 ± 8.3	***p* = 0.0006**
PFIQ-7 bowel/rectum (range 100-0)	11.6 ± 22.7	1.8 ± 4.8	***p* = 0.0116**
PFIQ-7 vagina/pelvis (range 100-0)	5.7 ± 12.9	0.9 ± 3.5	***p* = 0.0011**

Acronyms: Data are presented as median (IQR), if not stated otherwise. Statistically significant differences (p<0.05) are marked in bold. The number of respondents to the SFQ-28 questionnaire was 61 for SSc patients and 75 for healthy women; IQR, inter-quartile range; SD, standard deviation; SSc, systemic sclerosis; FSFI, Female Sexual Function Index; BISF-W, Brief Index of Sexual Function for Women; SFQ-28; Sexual Function Questionnaire; SQoL-F, Sexual Quality of Life - Female; PISQ-12, Pelvic Organ Prolapse/Urinary Incontinence Sexual Questionnaire short form; PFIQ-7, Pelvic Floor Impact Questionnaire – short form 7.

Table S2. Spearman's and Pearson's* correlation coefficients of sexual function and pelvic floor function with disease-related laboratory and clinical features SSc patients

		BISF-W D1	BISF-W D2	BISF-W D3	BISF-W D4	BISF-W D5	BISF-W D6	BISF-W D7	SFQ28 Desire	SFQ28 AS	SFQ28 AL	SFQ28 AC	SFQ28 Orgasm	SFQ28 Pain	SFQ28 Enjoyment	SFQ28 Partner
ESSG	r	**-0.057**	**-0.256**	**-0.254**	**-0.314**	**-0.243**	**-0.256**	0.064	**-0.319**	-0.242	-0.207	-0.123	0.003	-0.189	-0.240	-0.034
	p	**0.600**	**0.017**	**0.018**	**0.003**	**0.023**	**0.017**	0.555	**0.012**	0.061	0.109	0.351	0.983	0.146	0.063	0.792
	n	**87**	**87**	**87**	**87**	**87**	**87**	87	**61**	61	61	61	61	61	61	61
ESR	r	**-0.245**	**-0.296**	**-0.311**	**-0.274**	**-0.256**	-0.170	**-0.236**	-0.189	-0.147	-0.205	-0.109	0.001	0.159	-0.172	0.080
	p	**0.006**	**0.006**	**0.004**	**0.012**	**0.019**	0.121	**0.030**	0.152	0.267	0.120	0.415	0.993	0.229	0.193	0.545
	n	**84**	**84**	**84**	**84**	**84**	84	**84**	59	59	59	59	59	59	59	59
Dyspnea	r	**-0.286***	**-0.273***	**-0.270***	**-0.226***	**-0.317***	-0.149*	0.083*	-0.230*	-0.212*	-0.243*	**-0.267***	-0.169*	-0.050*	-0.069*	0.073*
	p	**0.008**	**0.011**	**0.012**	**0.036**	**0.003**	0.170	0.447	0.077	0.104	0.062	**0.041**	0.197	0.702	0.603	0.581
	n	**86**	**86**	**86**	**86**	**86**	86	86	60	60	60	**60**	60	60	60	60
ILD	r	**-0.367***	**-0.334***	**-0.292***	**-0.247***	**-0.300***	**-0.290***	0.093*	-0.219*	-0.025*	-0.140*	-0.057*	-0.114*	-0.117*	0.001*	0.030*
	p	**0.001**	**0.002**	**0.007**	**0.023**	**0.005**	**0.007**	0.398	0.095	0.852	0.289	0.673	0.391	0.901	0.991	0.820
	n	**85**	**85**	**85**	**85**	**85**	**85**	85	59	59	59	59	59	59	59	59
DLCO	r	**0.301**	**0.383**	**0.396**	**0.380**	**0.361**	**0.300**	0.157	0.187	0.086	0.231	0.171	0.174	0.152	0.247	0.065
	p	**0.007**	**0.001**	**0.001**	**0.001**	**0.001**	**0.007**	0.164	0.168	0.529	0.086	0.212	0.201	0.265	0.066	0.633
	n	**80**	**80**	**80**	**80**	**80**	**80**	80	56	56	56	56	56	56	56	56
Dysphagia/ pyrosis	r	-0.138	-0.175	-0.175	-0.071	-0.151	-0.183	-0.014	-0.173	-0.042	-0.037	-0.039	-0.221	0.067	-0.095	0.047
	p	0.205	0.107	0.106	0.517	0.165	0.092	0.899	0.186	0.749	0.780	0.768	0.089	0.611	0.471	0.722
	n	86	86	86	86	86	86	86	60	60	60	60	60	60	60	60
Obstipation/ dyarrhea	r	**-0.264**	-0.188	-0.144	-0.176	-0.163	-0.196	0.084	-0.094	-0.118	-0.188	**-0.270**	-0.206	0.014	-0.105	-0.102
	p	**0.015**	0.085	0.187	0.106	0.136	0.072	0.447	0.479	0.374	0.155	**0.040**	0.117	0.915	0.430	0.441
	n	**85**	85	85	85	85	85	85	59	59	59	**59**	59	59	59	59
Antidepressants	r	**-0.227***	**-0.370***	-0.216*	**-0.245***	**-0.361***	**-0.218***	0.113*	**-0.319***	**-0.281***	-0.221*	-0.187*	**-0.398***	-0.244*	**-0.303***	**-0.454***
	p	**0.047**	**0.001**	0.060	**0.032**	**0.001**	**0.050**	0.327	**0.016**	**0.036**	0.101	0.168	**0.002**	0.070	**0.023**	**0.001**
	n	**77**	**77**	77	**77**	**77**	**77**	77	**56**	**56**	56	56	**56**	56	**56**	**56**
SSc limitations	r	**-0.425***	**-0.415***	**-0.382***	**-0.357***	**-0.339***	**-0.552***	**0.393***	**-0.473***	**-0.278***	**-0.301***	-0.232*	**-0.326***	**-0.326***	**-0.370***	**-0.328***
	p	**0.002**	**0.002**	**0.005**	**0.009**	**0.013**	**0.001**	**0.004**	**0.001**	**0.049**	**0.032**	0.102	**0.020**	**0.019**	**0.008**	**0.019**
	n	**53**	**53**	**53**	**53**	**53**	**53**	**53**	**51**	**51**	**51**	51	**51**	**51**	**51**	**51**
Education	r	**0.212***	**0.288***	**0.234***	**0.264***	**0.250***	0.153*	-0.060*	0.181*	0.152*	**0.254***	0.092*	0.038*	0.060*	0.237*	0.112*
	p	**0.050**	**0.007**	**0.030**	**0.014**	**0.020**	0.159	0.585	0.163	0.242	**0.049**	0.484	0.773	0.644	0.066	0.390
	n	**86**	**86**	**86**	**86**	**86**	86	86	61	61	**61**	61	61	61	61	61
Alcohol	r	**0.396**	**0.453**	**0.791**	**0.432**	**0.420**	**0.363**	0.142	**0.314**	**0.269**	**0.431**	**0.290**	**0.289**	0.029	0.402	-0.063
	p	**0.001**	**0.001**	**0.001**	**0.001**	**0.001**	**0.001**	0.217	**0.018**	**0.045**	**0.001**	**0.030**	**0.031**	0.849	0.002	0.647
	n	**77**	**77**	**77**	**77**	**77**	**77**	77	**56**	**56**	**56**	**56**	**56**	56	56	56
SHAQ global	r	-0.176	**-0.320**	**-0.268**	-0.225	**-0.274**	**-0.291**	0.179	**-0.369**	**-0.446**	-0.232	**-0.380**	**-0.363**	**-0.414**	**-0.438**	**-0.424**
	p	0.131	**0.005**	**0.020**	0.053	**0.017**	**0.011**	0.125	**0.008**	**0.001**	0.105	**0.007**	**0.010**	**0.003**	**0.001**	**0.002**
	n	75	**75**	**75**	75	**75**	**75**	75	**50**	**50**	50	**50**	**50**	**50**	**50**	**50**
HAQ	r	-0.161	**-0.258**	**-0.243**	**-0.245**	**-0.247**	**-0.305**	0.162	**-0.342**	**-0.325**	-0.177	**-0.274**	-0.239	**-0.350**	**-0.336**	**-0.309**
	p	0.139	**0.016**	**0.024**	**0.023**	**0.022**	**0.004**	0.137	**0.008**	**0.011**	0.177	**0.036**	0.066	**0.006**	**0.009**	**0.016**
	n	86	**86**	**86**	**86**	**86**	**86**	60	**60**	**60**	60	**60**	60	**60**	**60**	**60**
BDI-II	r	**-0.387**	**-0.446**	**-0.438**	**-0.352**	**-0.398**	**-0.425**	**0.279**	**-0.455**	**-0.312**	**-0.349**	**-0.440**	-0.211	**-0.303**	**-0.488**	**-0.541**
	p	**0.001**	**0.001**	**0.001**	**0.001**	**0.001**	**0.001**	**0.009**	**0.001**	**0.015**	**0.006**	**0.001**	0.106	**0.018**	**0.001**	**0.001**
	n	**86**	**86**	**86**	**86**	**86**	**86**	**86**	**60**	**60**	**60**	**60**	60	**60**	**60**	**60**
FIS	r	**-0.358**	**-0.453**	**-0.397**	**-0.365**	**-0.438**	**-0.437**	0.187	**-0.401**	**-0.377**	**-0.321**	**-0.420**	**-0.259**	-0.228	**-0.540**	**-0.373**
	p	**0.001**	**0.001**	**0.001**	**0.001**	**0.001**	**0.001**	0.082	**0.001**	**0.003**	**0.012**	**0.001**	**0.044**	0.078	**0.001**	**0.003**
	n	**87**	**87**	**87**	**87**	**87**	**87**	87	**61**	**61**	**61**	**61**	**61**	61	**61**	**61**
HAP-AAS	r	**0.451**	**0.524**	**0.531**	**0.445**	**0.447**	**0.502**	0.010	**0.311**	**0.339**	**0.316**	**0.361**	0.135	**0.286**	**0.433**	0.168
	p	**0.001**	**0.001**	**0.001**	**0.001**	**0.001**	**0.001**	0.926	**0.016**	**0.008**	**0.014**	**0.005**	0.302	**0.027**	**0.001**	0.199
	n	**86**	**86**	**86**	**86**	**86**	**86**	86	**60**	**60**	**60**	**60**	60	**60**	**60**	60
SF-36 PCS	r	**0.258**	**0.310**	**0.318**	**0.246**	**0.308**	**0.294**	-0.120	**0.322**	**0.404**	**0.275**	**0.355**	0.204	**0.335**	**0.421**	0.188
	p	**0.017**	**0.004**	**0.003**	**0.023**	**0.004**	**0.008**	0.272	**0.012**	**0.001**	**0.033**	**0.006**	0.118	**0.009**	**0.001**	0.150
	n	**85**	**85**	**85**	**85**	**85**	**85**	85	**60**	**60**	**60**	**60**	60	**60**	**60**	60
SF-36 MCS	r	**0.239**	**0.352**	**0.274**	0.207	**0.295**	**0.269**	**-0.235**	**0.275**	**0.262**	**0.282**	**0.299**	0.186	0.222	**0.401**	**0.379**
	p	**0.027**	**0.001**	**0.011**	0.057	**0.006**	**0.013**	**0.030**	**0.034**	**0.043**	**0.029**	**0.021**	0.154	0.089	**0.002**	**0.003**
	n	**85**	**85**	**85**	85	**85**	**85**	**85**	**60**	**60**	**60**	**60**	60	60	**60**	**60**

Acronyms: Statistically significant correlations ($p<0.05$) are marked in bold. Pearson's correlation coefficients are marked with *. SSc, systemic sclerosis; ESSG, European Scleroderma Study Group; ESR, erythrocyte sedimentation rate; ILD, interstitial lung disease; DLCO, diffusing capacity of the lungs for carbon monoxide; SSc limitations, the presence of difficulties associated with systemic sclerosis limiting sexual activity; HAQ, Health Assessment Questionnaire; SHAQ Global, Scleroderma Health Assessment Questionnaire – Global Score, aggregated score of HAQ and SHAQ; HAP AAS, Human Activity Profile Adjusted Activity Score; FIS, Fatigue Impact Scale; BDI-II, Beck's Depression Inventory-II; SF-36 MCS, Medical outcomes study Short Form 36 - Mental Component Summary; SF-36 PCS, Medical outcomes study Short Form 36 - Physical Component Summary; FSFI, Female Sexual Function Index; BISF-W, Brief Index of Sexual Function for Women; SQoL-F, Sexual Quality of Life - Female; PFIQ-7, Pelvic Floor Impact Questionnaire – short form 7; PISQ-12, Pelvic Organ Prolapse/Urinary Incontinence Sexual Questionnaire short form.

Supplementary Table S3: Multivariate regression analysis predicting sexual function and pelvic floor function in female patients with SSc based on clinical features

	β (95% CI)	Stand. β	*p*	Adjusted R^2	*p* *
**FSFI**					
Overall model				0.178	**0.017**
Disease duration	-0.266 (-0.686; 0.154)	-0.161	0.210		
mRSS	0.116 (-0.175; 0.407)	0.110	0.426		
ESSG activity index	-1.914 (-4.041; 0.212)	-0.254	0.077		
ESR	-0.029 (-0.187; 0.129)	-0.053	0.710		
DLCO	0.047 (-0.120; 0.214)	0.090	0.575		
SHAQ-Global	-7.183 (-12.301; -2.066)	-0.338	**0.007**		
dysphagia/pyrosis	-1.512 (-7.641; 4.617)	-0.065	0.623		
obstipation/diarhea	-2.026 (-8.425; 4.373)	-0.077	0.528		
PAH	6.503 (-0.624; 13,631)	0.229	0.073		
**BISF-W**					
Overall model				0.148	**0.003**
Disease duration	-0.133 (-0.796; 0.530)	-0.052	0.688		
mRSS	0.359 (-0.100; 0.817)	0.218	0.123		
ESSG activity index	-3.287 (-6.661; 0.087)	-0.277	0.056		
ESR	-0.166 (-0.411; 0.078)	-0.195	0.178		
DLCO	0.025 (-0.241; 0.291)	0.030	0.852		
SHAQ-Global	-9.713 (-17.629; -1.797)	-0.300	**0.017**		
dysphagia/pyrosis	0.241 (-9.507; 9.989)	0.007	0.961		
obstipation/diarhea	-6.871 (-17.010; 3.268)	-0.170	0.180		
PAH	5.299 (-5.976; 16.573)	0.121	0.350		
**SQoL-F**					
Overall model				0.376	**<0.0001**
Disease duration	0.002 (-0.917; 0.920)	0.000	0.997		
mRSS	0.863 (0.213; 1.514)	0.330	**0.010**		
ESSG activity index	-9.561 (-14.270; -4.851)	-0.507	**<0.0001**		
ESR	0.069 (-0.274; 0.412)	0.052	0.689		
DLCO	-0.023 (-0.404; 0.358)	-0.018	0.903		
SHAQ-Global	-26.768 (-37.934; -15.601)	-0.521	**<0.0001**		
dysphagia/pyrosis	-0.658 (-14.118; 12.803)	-0.012	0.922		
obstipation/diarhea	-1.541 (-15.836; 12.754)	-0.024	0.829		
PAH	10.814 (-4.759; 26.387)	0.159	0.169		
**PISQ-12**					
Overall model				0.179	**0.004**
Disease duration	0.006 (-0.213; 0.225)	0.007	0.957		
mRSS	-0.169 (-0.338; 0.001)	-0.304	0.051		
ESSG activity index	1.697 (0.501; 2.894)	0.438	**0.006**		
ESR	-0.017 (-0.098; 0.065)	-0.061	0.683		
DLCO	0.016(-0.080; 0.111)	0.058	0.744		
SHAQ-Global	2.904 (0.211; 5.597)	0.275	**0.035**		
dysphagia/pyrosis	1.169 (-2.079; 4.417)	0.100	0.473		
obstipation/diarhea	4.237 (0.851; 7.622)	0.319	**0.015**		
PAH	0.321 (-3.629; 4.270)	0.022	0.871		
**PFIQ-7**					
Overall model				0.057	0.060
Disease duration	-0.477 (-2.544; 1.589)	-0.062	0.645		
mRSS	-0.795 (-2.222; 0.632)	-0.161	0.269		
ESSG activity index	0.693 (-9.756; 11.143)	0.020	0.895		
ESR	-0.699 (-1.463; 0.064)	-0.273	0.072		
DLCO	-0.862 (-1.686; -0.037)	-0.354	**0.041**		
SHAQ-Global	16.972 (-7.721; 41.666)	0.174	0.174		

dysphagia/pyrosis	9.051 (-20.988; 39.090)	0.084	0.548
obstipation/diarhea	7.254 (-23.109; 37.616)	0.061	0.634
PAH	-16.010 (-51.150; 19.130)	-0.121	0.365

Acronyms: Statistical significance ($p<0.05$) is marked in bold. β, regression beta coefficient; stand. β, standardized regression beta coefficient; CI, confidence interval; p, p-value of the predictor in the model; Adjusted R^2, R-squared adjusted for the number of predictors in the model; *p* *, *p*-value for the overall model; SSc, systemic sclerosis; FSFI, Female Sexual Function Index; BISF-W, Brief Index of Sexual Function for Women; SQoL-F, Sexual Quality of Life – Female; PISQ-12, Pelvic Organ Prolapse/Urinary Incontinence Sexual Questionnaire short form; PFIQ-7, Pelvic Floor Impact Questionnaire – short form 7; ESSG, European Scleroderma Study Group; ESR, erythrocyte sedimentation rate; mRSS, modified Rodnan Skin Score; DLCO, diffusing capacity of the lungs for carbon monoxide; PAH, pulmonary arterial hypertension; SHAQ Global, Scleroderma Health Assessment Questionnaire – Global Score; SHAQ, Scleroderma Health Assessment Questionnaire – a total score of five visual analog scales; FIS, Fatigue Impact Scale, HAP AAS, Human Activity Profile Adjusted Activity Score.

Supplementary Table S4. Sexual function and pelvic floor function in SSc patients with low and high disease activity assessed by European Scleroderma Study Group Activity Index

Parameters (score range worst-best)	SSc ESSG ≤ 3 (n = 68)	SSc ESSG > 3 (n = 22)	*p-value*
**FSFI total (range 2-36)**	19.4 (3.9-29.6)	13.8 (4.2-22.4)	*p = 0.181*
FSFI desire (range 1.2-6)	2.4 (1.2-3.6)	2.4 (1.7-3.6)	*p = 0.992*
FSFI arousal (range 0-6)	3.0 (1.2-4.8)	1.5 (0.0-3.6)	***p = 0.024***
FSFI lubrication (range 0-6)	3.6 (0.3-5.4)	1.7 (0.0-5.1)	*p = 0.137*
FSFI orgasm (range 0-6)	3.2 (0.0-5.2)	2.6 (0.0-3.8)	*p = 0.202*
FSFI satisfaction (range 0.8-6)	3.6 (1.2-5.2)	1.8 (0.8-4.1)	*p = 0.068*
FSFI pain (range 0-6)	3.6 (0.0-6.0)	1.0 (0.0-5.2)	*p = 0.183*
**BISF-W total (range -16-75)**	18.5 (3.4-35.7)	7.2 (0.3-27.5)	*p = 0.092*
BISF-W thoughts/desire (range 0-12)	3.1 (0.6-5.9)	4.1 (1.1-5.7)	*p = 0.747*
BISF-W arousal (range 0-12)	4.3 (0.4-7.4)	0.9 (0.0-6.3)	*p = 0.126*
BISF-W frequency of sexual activity (range 0-12)	1.8 (0.4-4.3)	0.8 (0.0-3.0)	*p = 0.185*
BISF-W receptivity/initiation (range 0-15)	6.0 (0.0-9.5)	2.0 (0.0-9.0)	*p = 0.145*
BISF-W pleasure/orgasm (range 0-12)	3.3 (0.2-6.3)	0.6 (0.0-3.5)	***p = 0.023***
BISF-W relationship satisfaction (range 0-12)	6.0 (3.0-9.5)	4.0 (1.8-7.0)	***p = 0.046***
BISF-W problems affecting sexual function (range 16-0)	4.2 (3.0-6.6)	4.6 (3.6-6.7)	*p = 0.451*
SFQ-28 desire (range 5-31)	17.0 (12.0-20.0)	16.0 (11.5-17.8)	*p = 0.406*
SFQ-28 arousal sensation (range 4-20)	10.0 (8.5-13.5)	8.0 (5.0-12.0)	*p = 0.080*
SFQ-28 arousal lubrication (range 2-10)	5.0 (4.0-7.0)	6.0 (2.5-7.0)	*p = 0.864*
SFQ-28 arousal cognitive (range 2-10)	5.0 (4.0-6.0)	5.0 (2.5-6.0)	*p = 0.300*
SFQ-28 orgasm (range 1-15)	10.0 (6.0-12.0)	10.0 (6.3-11.0)	*p = 0.844*
SFQ-28 pain (range 2-15)	13.0 (10.0-15.0)	11.0 (7.5-14.3)	*p = 0.303*
SFQ-28 enjoyment (range 6-30)	19.0 (14.5-24.0)	15.0 (9.0-22.5)	*p = 0.271*
SFQ-28 partner (range 2-10)	9.0 (8.0-10.0)	9.0 (5.8-10.0)	*p = 0.751*
**SQoL-F (range 0-100)**	64.4 (37.8-92.2)	48.3 (32.5-62.2)	***p = 0.032***
**PISQ-12 (range 48-0)**	11.0 (8.0-16.0)	15.5 (11.5-19.0)	***p = 0.010***
**PFIQ-7 total (range 300-0), mean±SD**	30.7 ± 47.4	35.3 ± 51.9	*p = 0.532*
PFIQ-7 bladder/urine (range 100-0)	14.8 ± 24.7	13.6 ± 20.5	*p = 0.791*
PFIQ-7 bowel/rectum (range 100-0)	10.4 ± 22.9	15.2 ± 22.4	*p = 0.203*
PFIQ-7 vagina/pelvis (range 100-0)	5.5 ± 11.4	5.5 ± 17.0	*p = 0.819*

Acronyms: Data are presented as median (IQR), if not stated otherwise. Statistically significant differences ($p<0.05$) are marked in bold. The number of respondents to the SFQ-28 questionnaire was 49 for SSc patients with ESSG ≤ 3 and 12 for SSc patients with ESSG > 3; ESSG, European Scleroderma Study Group Activity Index,; IQR, inter-quartile range; SD, standard deviation; SSc, systemic sclerosis; FSFI, Female Sexual Function Index; BISF-W, Brief Index of Sexual Function for Women; SFQ-28; Sexual Function Questionnaire; SQoL-F, Sexual Quality of Life - Female; PISQ-12, Pelvic Organ Prolapse/Urinary Incontinence Sexual Questionnaire short form; PFIQ-7, Pelvic Floor Impact Questionnaire – short form 7.

SUPPLEMENTARY REFERENCES

1. World Health Organization, Canada CPHAo. Preventing chronic diseases: a vital investment: World Health Organization; 2005.
2. Steen VD, Medsger Jr TA. The value of the Health Assessment Questionnaire and special patient-generated scales to demonstrate change in systemic sclerosis patients over time. Arthritis Rheum. 1997;40(11):1984-91.
3. Georges C, Chassany O, Mouthon L, Tiev K, Toledano C, Meyer O, et al. Validation of French version of the scleroderma health assessment questionnaire (SSc HAQ). Clin Rheumatol. 2005;24(1):3-10.
